# Estetrol and its implications for contraception, venous thromboembolism, and breast cancer: an integrative review

**DOI:** 10.1590/1677-5449.20260012

**Published:** 2026-08-28

**Authors:** Marcos Arêas Marques, Suzanna Maria Viana Sanches, Monique Magnavita Borba da Fonseca Cerqueira, Marina Araujo Zulchner, André Luiz Malavasi Longo de Oliveira

**Affiliations:** 1 Universidade Federal do Estado do Rio de Janeiro – UNIRIO, Hospital Universitário Gaffrée e Guinle, Rio de Janeiro, RJ, Brasil.; 2 Universidade do Estado do Rio de Janeiro – UERJ, Hospital Universitário Pedro Ernesto – HUPE, Rio de Janeiro, RJ, Brasil.; 3 Hospital Geral Ernesto Simões – HGES, Salvador, BA, Brasil.; 4 Universidade do Estado da Bahia – UNEB, Salvador, BA, Brasil.; 5 Universidade de São Paulo – USP, Faculdade de Medicina, São Paulo, SP, Brasil.

**Keywords:** deep vein thrombosis, pulmonary embolism, estetrol, combined oral contraceptives, breast cancer, estrogens

## Abstract

Combined oral contraceptives (COCs) provide benefits beyond contraception but are associated with risks such as venous thromboembolism (VTE) and breast cancer. This integrative review aimed to evaluate the efficacy and safety of estetrol (E4)-containing COCs regarding their risk of VTE and breast cancer. The PubMed database was searched for preclinical and clinical studies published through 2025. Data extraction followed a structured protocol, resulting in the inclusion of 15 studies in the review. Available evidence suggests that E4 has limited effects on the liver and the coagulation cascade, in addition to being associated with less breast tissue proliferation compared to other estrogens. However, clinical evidence regarding hard outcomes, including the long-term incidence of VTE and breast cancer, remains limited. Current evidence suggests a promising efficacy and safety profile but remains insufficient to rule out the risks of VTE and breast cancer.

## INTRODUCTION

Combined oral contraceptives (COCs) are associated with high adherence rates because they provide benefits beyond contraception, including reduced menstrual bleeding and dysmenorrhea, as well as improvement of premenstrual syndrome, acne, and hirsutism. In addition, COCs offer long-term benefits, including a reduced incidence of endometrial, ovarian, and colorectal cancers.

However, it is important to emphasize that, although rare, COCs are associated with significant adverse effects, such as venous thromboembolism (VTE) and breast cancer (BC).^1^ COCs increase the risk of VTE from a baseline of 5 cases per 10,000 women-years among nonusers to approximately 9 to 10 cases per 10,000 women-years among users, varying according to the type and dose of estrogen and progestogen included in the formulation. To place these figures into context, the incidence of VTE is approximately 29 cases per 10,000 women-years during pregnancy and increases to 300-400 cases per 10,000 women-years during the postpartum period. These data indicate that, in certain clinical situations, indiscriminate contraindication of COC use based solely on concerns about VTE risk may not be justified.^1^

The risk of VTE in women using COCs is related to hemostatic changes induced primarily by estrogens, which increase serum levels of coagulation factors (prothrombin; factors VII, VIII, and X; and fibrinogen) and decrease levels of anticoagulant factors (protein S and antithrombin), in addition to potentially inducing activated protein C resistance. First-generation COCs containing more than 50 μg of ethinyl estradiol (EE) are associated with a higher risk of VTE than later-generation COCs containing less than 50 μg of EE. However, there is no conclusive evidence of further risk reduction with COCs containing 20 μg of EE compared with those containing 30 μg. Although COCs containing less than 35 μg of EE are associated with a lower incidence of estrogen-related adverse effects, such as nausea and breast tenderness during the first months of use, this reduction does not extend to VTE risk.^1^ In addition, VTE risk is also influenced by individual factors, including a personal or family history of VTE, increasing body weight and age, thrombophilia, and the resumption or switching of COCs after an interruption of more than 4 weeks.

Another determinant of VTE risk is the type of progestogen used. Second-generation progestogens, such as levonorgestrel (LNG) and norethisterone, are considered safer than third- and fourth-generation progestogens. Although COCs containing third-generation progestogens are associated with fewer androgenic effects and possibly a lower cardiometabolic risk, they may be associated with a higher risk of VTE.^1^ Conversely, hemostasis is not significantly altered by progestogen-only contraceptives, LNG implants, or depot medroxyprogesterone acetate injections.^1^

COC use is an established risk factor for BC and is associated with a slight increase in risk that returns to baseline approximately 10 years after discontinuation.^2^ Although the increase in individual risk is modest, the widespread use of these methods makes their population-level impact relevant, corresponding to an estimated 13 additional cases per 100,000 women-years. However, despite the statistically significant relative increase in risk, the absolute risk increase is small and should be considered in the context of the well-established benefits of hormonal contraceptives, including the prevention of unintended pregnancies, which are associated with higher maternal morbidity and mortality, and protection against ovarian and endometrial cancers.^3^

Estetrol (E4) is an estrogen produced by the human fetal liver during pregnancy and has been developed for use in COCs in combination with drospirenone (DRSP) and in menopausal hormone therapy (MHT) for the management of menopausal symptoms.^4^

The objective of this review was to assess the efficacy and safety of E4 in contraception and MHT, particularly regarding its effect on the risk of VTE and BC.

## METHODS

This integrative literature review synthesized evidence from preclinical studies, phase I, II, and III clinical trials, pharmacodynamic studies, and safety analyses published through 2025 regarding (1) the efficacy and safety of E4 in contraception; (2) the effects of E4 on breast tissue and its potential implications for BC development; and (3) the effects of E4 on hemostasis and the risk of VTE.

The review was conducted in six stages: (1) formulation of the research question; (2) literature search and study selection; (3) study categorization; (4) methodological assessment; (5) interpretation of findings; and (6) presentation of the evidence synthesis. The research question was developed using the PICO framework (population/problem, intervention, comparison, and outcome): “What evidence is available regarding the efficacy and safety of E4 in contraception and its effects on breast tissue and hemostasis, including potential implications for the risks of BC and VTE?”.

The PubMed database was searched using the controlled descriptor “estetrol” (MeSH and DeCS). Randomized clinical trials and cohort studies addressing the research question and published in English, Portuguese, or Spanish were included. The results were organized in a Microsoft Excel® spreadsheet. Data extraction was performed using a structured summary table containing the title, authors, population and intervention, study design, duration, sample size, safety outcomes, and main findings. Two reviewers independently performed the data extraction.

## RESULTS

**Figure 1:**
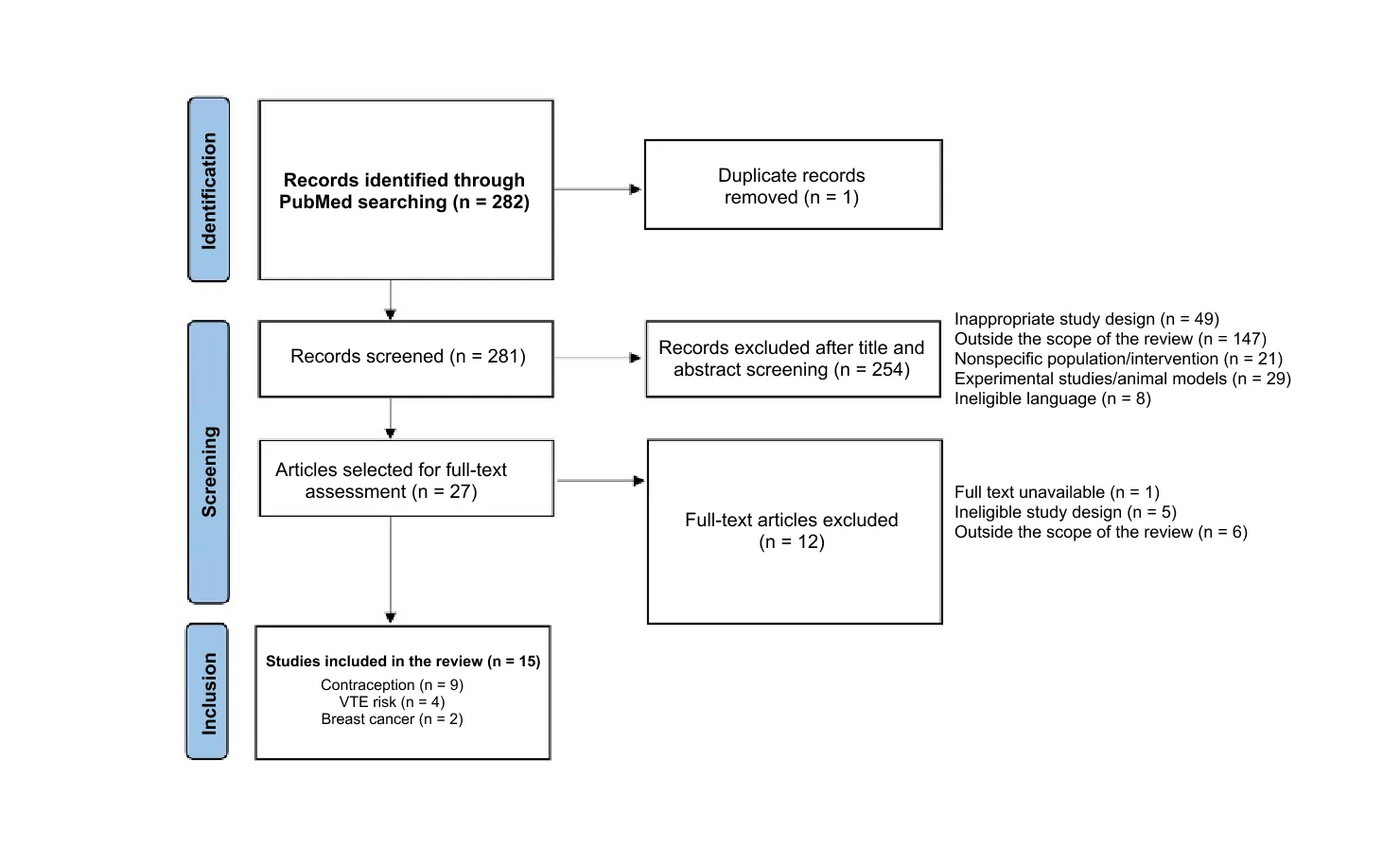
Flow diagram of study selection. VTE = venous thromboembolism.

A total of 282 articles were identified through the literature search. After removal of one duplicate record, 281 articles underwent title and abstract screening. Of these, 254 were excluded, leaving 27 studies for full-text assessment. Following full-text review, 15 studies met the eligibility criteria and were included in the review (Tables 1, 2 and 3). The study selection flow diagram is presented in Figure 1.

**Table 1:** Summary of studies included in the integrative review that assessed the contraceptive efficacy of E4.

**Author/year**	**Number of participants**	**Intervention**	**Comparator regimen**	**Outcomes**	**Conclusion**	**Level of evidence** *
Creinin et al., 2021^4^	1864	Open-label, multicenter, phase III study; E4 15 mg + DRSP 3 mg.	—	Contraceptive efficacy (Pearl Index); cycle control: vaginal bleeding pattern; safety: adverse events.	E4/DRSP was effective as an oral contraceptive, with a predictable bleeding pattern for most women and low rates of adverse events.	2b
Kipling et al., 2021^5^	98	Randomized, controlled, open-label, 3-arm parallel study (4:3:3); E4 15 mg/DRSP 3 mg vs EE/LNG vs EE/DRSP.	Comparator 1: EE 30 μg/LNG 150 μg. Comparator 2: EE 20 μg/DRSP 3 mg.	Effect of E4 15 mg/DRSP 3 mg on endocrine and metabolic parameters after 3 and 6 months of treatment.	E4 15 mg/DRSP 3 mg showed limited effects on metabolic and endocrine parameters, with less pronounced effects on gonadotropins, cortisol, CBG, angiotensinogen, SHBG, and triglycerides than EE-containing formulations.	1b
Gemzell‐Danielsson et al., 2021^6^	1553	Open-label, multicenter, phase III study; E4/DRSP in a 24/4 regimen for up to 13 28-day cycles.	—	Contraceptive efficacy (Pearl Index); bleeding pattern; safety.	E4/DRSP demonstrated contraceptive efficacy, a predictable bleeding pattern, and a favorable safety profile.	2b
Duijkers et al., 2021^7^	82	Randomized, open-label, single-center, parallel study; E4 15 mg/DRSP 3 mg (24/4) for three consecutive cycles.	EE 20 µg/DRSP 3 mg.	Primary: suppression of ovarian function (Hoogland score); secondary: pituitary-ovarian function, endometrial thickness, and return of ovulation.	No participant in the E4/DRSP group ovulated, whereas two participants ovulated in the EE/DRSP group; findings suggest adequate inhibition of ovulation and suppression of ovarian function (exploratory study).	1b
Apter et al., 2017^8^	396	Randomized, open-label, multicenter, dose-finding study; six cycles (24/4) in five groups: (1) E4 15 mg/DRSP 3 mg; (2) E4 15 mg/LNG 150 μg; (3) E4 20 mg/DRSP 3 mg; (4) E4 20 mg/LNG 150 μg; (5) E2V/DNG.	E2V/DNG (Qlaira^®^)	Primary: bleeding pattern/cycle control (E4+DRSP or E4+LNG); secondary: satisfaction, well-being, and acceptability.	E4 15 mg/DRSP 3 mg was associated with high acceptability and satisfaction, as well as a favorable body weight-control profile.	1b
Apter et al., 2016^11^	396	Same intervention as the dose-finding study^8^; six cycles (24/4) in five groups: (1) E4 15 mg/DRSP 3 mg; (2) E4 15 mg/LNG 150 μg; (3) E4 20 mg/DRSP 3 mg; (4) E4 20 mg/LNG 150 μg; (5) E2V/DNG.	E2V/DNG (Qlaira^®^)	Primary: vaginal bleeding patterns and cycle control.	Among the four E4-containing combinations evaluated, E4 15 mg/DRSP 3 mg showed the most favorable vaginal bleeding and cycle control profile.	1b
Duijkers et al., 2015^10^	108	Open-label, parallel, phase II, dose-finding, pilot study; three cycles (24/4 days) in six groups: (1) E4 5 mg/DRSP 3 mg; (2) E4 10 mg/DRSP 3 mg; (3) EE 20 μg/DRSP 3 mg; (4) E4 5 mg/LNG 150 μg; (5) E4 10 mg/LNG 150 μg; (6) E4 20 mg/LNG 150 μg.	EE 20 µg/DRSP 3 mg.	Ovulation rates (Hoogland score).	When combined with a progestin, E4 adequately suppresses ovarian activity, particularly at doses ≥ 10 mg/day.	2b
Mawet et al., 2015^12^	109	Dose-finding, single-center, exploratory, controlled study; three cycles (24/4 days) in six groups: (1) E4 5 mg/DRSP 3 mg; (2) E4 10 mg/DRSP 3 mg; (3) E4 5 mg/LNG 150 μg; (4) E4 10 mg/LNG 150 μg; (5) E4 20 mg/LNG 150 μg; (6) EE 20 μg/DRSP 3 mg.	EE 20 µg/DRSP 3 mg.	Hepatic and metabolic parameters, bone markers, and growth factors.	E4-containing combinations showed limited effects on liver function, lipid metabolism, and bone and growth endocrine parameters compared with EE-containing formulations.	2b
Kluft et al., 2016^9^	48	Open-label, parallel, dose-finding, single-center study; 24/4 regimen in three groups: (1) EE 20 μg/DRSP 3 mg (Yaz®); (2) E4 5 mg/DRSP 3 mg; (3) E4 10 mg/DRSP 3 mg.	EE 20 µg/DRSP 3 mg (Yaz^®^)	Markers of hepatic estrogenicity and hemostasis.	Findings suggest lower hepatic estrogenicity with E4/DRSP compared with EE/DRSP, generating the hypothesis of a lower potential risk of VTE.	2b

CBG = corticosteroid-binding globulin; DNG = dienogest; DRSP = drospirenone; E2V = estradiol valerate; E4 = estetrol; EE = ethinyl estradiol; LNG = levonorgestrel; SHBG = sex hormone-binding globulin; VTE = venous thromboembolism. 24/4 regimen: 24 consecutive days of active tablets followed by 4 days of placebo tablets.

* Level of evidence according to the Oxford Centre for Evidence-Based Medicine classification.

**Table 2:** Summary of studies included in the integrative review that assessed the safety of E4 in relation to breast cancer.

**Author/year**	**N**	**Intervention**	**Comparator regimen**	**Outcomes**	**Conclusion**	**Level of evidence** *
Singer et al., 2014^26^	30	Pilot randomized clinical trial: E4 20 mg vs placebo in the perioperative setting of early-stage breast cancer; preoperative and postoperative biopsies performed.	Placebo	Tumor proliferation and apoptosis markers: Ki-67, ERα, ERβ, Bax, Bcl-2; systemic IGF-1 levels.	E4 demonstrated antiestrogenic effects in breast cancer cells, with increased apoptosis, ERα downregulation, and decreased systemic IGF-1 levels, suggesting the safety of E4 in the evaluated setting.	1b
Schmidt et al., 2021^27^	12	Open-label, multicenter, phase IB/IIA pilot dose-escalation study (3 + 3 cohort design): oral E4 20, 40, or 60 mg daily for 12 weeks in patients with heavily treated advanced breast cancer.	—	Primary: DLT and estimation of the maximum recommended and optimal doses; secondary: safety, tolerability, quality of life related to estrogen-deficiency symptoms, and preliminary assessment of antitumor response.	High doses of E4 were reported to be safe and well tolerated over 12 weeks, with signs of antitumor activity and no DLT, suggesting a potentially favorable cardiovascular risk profile and the possibility of maintaining or improving quality of life.	4

DLT = dose-limiting toxicity; ERα = estrogen receptor α; ERβ = estrogen receptor β; E4 = estetrol; IGF-1 = insulin-like growth factor 1.

* Level of evidence according to the Oxford Centre for Evidence-Based Medicine classification.

**Table 3:** Summary of studies included in the integrative review that assessed the safety of E4 in relation to venous thromboembolism.

**Author/year**	**Number of participants**	**Intervention**	**Comparator regimen**	**Outcomes**	**Conclusion**	**Level of evidence** *
Douxfils et al., 2020^30^	101	Randomized clinical trial: combined oral contraceptive containing E4 15 mg + DRSP 3 mg administered for six 28-day cycles in healthy women.	EE 20 μg + DRSP 3 mg	Hemostasis parameters, including activated protein C resistance, prothrombin fragment 1+2 (F1+2), SHBG, and other thromboembolic risk markers, assessed after 3 and 6 cycles.	The effect of E4/DRSP on hemostasis parameters was smaller or similar to that of EE/LNG and lower than that of EE/DRSP, suggesting a more favorable hemostasis profile. The findings indicate a possible reduction in thromboembolic risk associated with the estrogen component.	1b
Kluft et al., 2017^9^	48	Open-label clinical trial: E4 (5 or 10 mg) + DRSP 3 mg administered for three cycles in healthy women.	EE 20 μg + DRSP 3 mg	Markers of hemostasis and hepatic estrogenicity (SHBG, angiotensinogen, APCr, D-dimer, and F1+2).	E4/DRSP showed a lower hemostatic impact and no apparent procoagulant effect compared with EE/DRSP, suggesting a potentially more favorable thrombotic profile.	2b
Kobayashi et al., 2024^28^	86	Multicenter randomized clinical trial: E4 15 mg + DRSP 3 mg administered for 12 weeks in patients with endometriosis.	EE 20 μg + DRSP 3 mg	Coagulation and fibrinolysis markers (APCsr/nAPCsr, D-dimer, F1+2, protein S, TFPI, and SHBG).	E4/DRSP had a lower impact on coagulation and fibrinolytic markers than EE/DRSP, suggesting a more favorable thrombotic profile, including in a population with a higher baseline risk of VTE.	1b
Douxfils et al., 2023^29^	180	Double-blind randomized clinical trial: E4 2.5–15 mg/day administered for 12 weeks.	Placebo	Hemostasis markers (nAPCsr, D-dimer, and F1+2), SHBG, lipid profile, glucose metabolism, and bone remodeling markers.	E4 had limited impact on hemostasis, with only minor and clinically irrelevant changes, and potentially beneficial effects in metabolic and bone parameters, suggesting a favorable thrombotic profile in postmenopausal women.	1b

APCr = activated protein C resistance; APCsr = activated protein C sensitivity ratio; DRSP = drospirenone; EE = ethinyl estradiol; E4 = estetrol; nAPCsr = normalized activated protein C sensitivity ratio; VTE = venous thromboembolism.

* Level of evidence according to the Oxford Centre for Evidence-Based Medicine classification.

### Efficacy and safety of E4 in contraception

Nine studies evaluating E4 for contraception, including cohort studies and randomized clinical trials, were identified.^4-12^

Early phase II studies^8-12^ evaluated different doses of E4 (5, 10, 15, and 20 mg) combined with DRSP 3 mg or LNG 150 μg, focusing on ovarian suppression, bleeding patterns, user acceptability, and metabolic and hepatic parameters. For comparison, some studies included formulations containing estradiol valerate (E2V) combined with dienogest (DNG) and EE 20 μg combined with DRSP 3 mg. In a pilot study, E4 doses greater than 10 mg, when combined with a progestin, demonstrated adequate ovarian suppression.^10^

The E4 15 mg/DRSP 3 mg combination showed high user acceptability and satisfaction and a favorable weight control profile. In a comparative analysis of different E4-containing combinations, the E4 15 mg/DRSP 3 mg combination also had the most favorable vaginal bleeding pattern and cycle control.^8,11^

In the multicenter, open-label, exploratory FIESTA study, 396 healthy women aged 18-35 years were treated for six cycles with one of five regimens: (1) E4 15 mg/DRSP 3 mg; (2) E4 20 mg/DRSP 3 mg; (3) E4 15 mg/LNG 150 μg; (4) E4 20 mg/LNG 150 μg; and (5) E2V/DNG (Qlaira®; comparator). The results of this trial were reported in two publications,^8,11^ both of which consistently showed that E4 15 mg/DRSP 3 mg was associated with high user acceptability and satisfaction, favorable body weight control, and a more favorable bleeding pattern than the other regimens evaluated.

In a randomized, open-label, single-center study, 82 participants were allocated in a 1:1 ratio to receive either E4 15 mg/DRSP 3 mg or EE 20 µg/DRSP 3 mg for three consecutive cycles. Outcomes included ovarian function suppression (expressed as Hoogland score), pituitary-ovarian function, endometrial thickness, and return of ovulation. No ovulation occurred in the E4 15 mg/DRSP 3 mg group, whereas two participants ovulated in the EE 20 μg/DRSP 3 mg group (one woman ovulated on two occasions within the same cycle), suggesting adequate ovulation inhibition with E4 15 mg/DRSP 3 mg.^7^

Another open-label, controlled, parallel study randomly assigned 98 participants, in a 4:3:3 ratio, to one of three treatment arms: (1) E4 15 mg/DRSP 3 mg; (2) EE 30 μg/LNG 150 μg; and (3) EE 20 μg/DRSP 3 mg. Endocrine and metabolic parameters were assessed after three and six cycles, including gonadotropins, sex hormone-binding globulin, cortisol, corticosteroid-binding globulin, angiotensinogen, and triglycerides, among others. Compared with the EE-containing formulations, E4 15 mg/DRSP 3 mg had only limited effects on these parameters.^5^

Based on these findings, the E4 15 mg/DRSP 3 mg regimen was selected for investigation in two pivotal phase III clinical trials (E4 FREEDOM), one conducted in Europe/Russia and the other in the United States/Canada. The trials evaluated contraceptive efficacy (Pearl Index [PI]), cycle control (bleeding pattern parameters according to Mishell criteria), and safety.^4,6^

The US/Canada study was conducted in 77 centers and included 1864 participants (16-50 years of age; body mass index [BMI] ≤ 30 kg/m^2^) who used E4 15 mg/DRSP 3 mg for 13 cycles (24/4 regimen). Contraceptive efficacy was assessed using the PI and method-failure PI; cycle control was evaluated according to bleeding pattern; and safety was assessed based on adverse events. The PI was 2.65 (95% CI, 1.73-3.88), and the method-failure PI was 1.43 (95% CI, 0.70-2.39). Scheduled bleeding was observed in 82.9% to 87.0% of participants per cycle, with a mean duration of 4.5 days, and unscheduled bleeding decreased after the initial cycles. The most frequent adverse events were headache (5.0%) and metrorrhagia (4.6%). No VTE events were reported.^4^

The Europe/Russia study was conducted in 69 centers and included 1553 participants (18-50 years of age; BMI ≤ 35 kg/m^2^) who used E4/DRSP for 13 cycles (24/4-day regimen). The primary outcome was contraceptive efficacy (PI) in the subgroup aged 18-35 years; secondary outcomes included the method-failure PI, cumulative pregnancy rates, bleeding patterns, and safety. A PI of 0.47 pregnancies per 100 women-years (95% CI, 0.15-1.11) and a method-failure PI of 0.29 pregnancies per 100 women-years (95% CI, 0.06-0.83) were reported in the subgroup aged 18-35 years. Scheduled bleeding/spotting occurred in 91.9% to 94.4% of participants per cycle, with a mean duration of 4-5 days, and unscheduled bleeding decreased throughout treatment (from 23.5% to < 16% within 6 months). The most common adverse events were headache (7.7%), metrorrhagia (5.5%), vaginal hemorrhage (4.8%), and acne (4.2%)^6^ (Table 1).

Pooled analyses derived from the phase III trials^13-16^ were not included in the body of evidence considered for this integrative review because they represented secondary analyses of previously reported study populations. Nevertheless, they provide relevant complementary information regarding clinically important subgroups and outcomes, including tolerability, bleeding patterns, safety, and efficacy in specific populations, and are therefore briefly discussed below.

An analysis of participants with cardiovascular (CV) risk factors found no significant differences in discontinuation rates between women with and without CV risk factors. Among more than 1400 users with CV risk factors, only 0.2% discontinued treatment because of hypertension, all of whom had high-normal blood pressure levels and at least one other CV risk factor.^13^

A pooled analysis of 3417 participants from the phase-3 trials reported favorable tolerability and no clinically relevant changes in body weight, BP, heart rate, or laboratory parameters during treatment.^14^ A subgroup efficacy analysis confirmed the high overall effectiveness of the regimen, with contraceptive failures being associated primarily with nonmodifiable factors (except adherence), such as Black race, younger age, and previous pregnancy.^15^ Finally, an assessment of bleeding patterns among 3409 participants found that 87.2% to 90.4% experienced scheduled bleeding or spotting per cycle, indicating a predictable pattern. Adherence and BMI influenced these outcomes.^16^

### E4 and breast cancer

The effects of estrogens are mediated primarily by estrogen receptors α (ERα) and β (ERβ), which act as transcription factors toward the regulation of target genes through the genomic pathway. In addition, estrogens can trigger rapid responses by activating membrane-initiated signaling through the G protein-coupled estrogen receptor (GPER), which is expressed in both normal and neoplastic cells, including BC cells.^17^ GPER-initiated signaling may induce the transcription of genes involved in processes such as cell proliferation, invasion, metastasis, angiogenesis, and tumor-promoting inflammation.^17^

The potential implications of E4 for BC may be considered in two contexts: (1) as a component of MHT, with a potentially more favorable BC risk profile; and (2) as a potential therapeutic strategy for established BC, based on antiestrogenic effects observed in specific experimental settings.

Current evidence indicates that MHT is associated with an increased risk of BC, particularly when estrogen is combined with a progestogen, suggesting the promotion of preexisting BC growth rather than cancer induction.^18,19^ Similarly, COCs are associated with a slight increase in this risk, which tends to return to baseline approximately 10 years after discontinuation, a pattern that is also consistent with the hypothesis of promotion of preexisting cancer growth.^19^

E4 exhibits affinity for both ERα and ERβ, although its behavior differs from that of other estrogens, showing a four- to five-fold greater affinity for ERα than for ERβ. Because of this distinctive profile, it has been classified as a native estrogen with selective tissue activity.^20^ It acts as an antagonist on membrane ERα and as an agonist on nuclear ERα, resulting predominantly in proestrogenic effects in bone, endometrium, vagina, and the cardiovascular system and antiestrogenic effects in breast tissue. These properties support the hypothesis that E4 may function as a selective estrogen receptor modulator, a concept supported by both *in vitro* and *in vivo* evidence, in addition to exhibiting lower binding affinity and estrogenic potency than 17β-estradiol (E2).^21^

Experimental studies have shown that E4 is associated with lower breast cell proliferation than E2, the primary biologically active estrogen in women of reproductive age. ER-positive BC models have shown E4 to promote mixed agonistic and antagonistic actions with the capacity to antagonize E2-induced cell proliferation and migration. E4 has also been shown to exhibit proapoptotic properties in endocrine-resistant BC cells, along with differential activation of signaling pathways, reduced induction of proliferative genes, and anti-invasive effects mediated through GPER and plasminogen activator inhibitor type 2.^18,22^ These findings further support the potential role of E4 in estrogen therapy for women at increased risk of BC and for patients with established disease.

The effects of E4, however, appear to be dose dependent. At high concentrations, protumoral and antiapoptotic effects have been described in estrogen-sensitive cells, although with lower potency than E2.^23^ Supratherapeutic doses, more than 10 times higher than those used in contraception and MHT studies, produced effects similar to those observed with therapeutic E2 in breast cells. Nevertheless, these findings have been interpreted as supporting a potentially more favorable safety profile for E4 under usual clinical conditions.^18,21^

Experimental studies since the 1980s have suggested that E4 is 50 to 100 times less potent than E2 in inducing progesterone receptors and may exhibit both agonistic and antagonistic properties under different conditions. Subsequent studies have indicated that E1, E2, E3, and E4 can stimulate tumor proliferation at high concentrations; however, at lower doses, the proliferative effect of E4 is substantially weaker than that of E2. The combination of E2 and E4 has also been evaluated in experimental models, with findings suggesting partial neutralization of E2-induced proliferative effects under certain conditions. This observation has been interpreted as evidence of antiestrogenic activity, although the underlying mechanisms remain incompletely understood. In addition, both *in vitro* and *in vivo* studies have demonstrated that E4 has a lesser impact on cell motility and invasion.^18,23-25^

The combination of E4 with a progestogen, proposed as a strategy for endometrial protection, has been evaluated in preclinical studies, with findings suggesting an overall neutral effect on the growth and dissemination of established BC. Within this body of evidence, E4 appears to have a more favorable profile than E2, and combinations with progestogens have not demonstrated a clear increase in protumoral activity. Transcriptomic analyses have also shown a limited impact of E4 combined with a progestogen on transcriptional pathways related to breast tissue growth, further supporting the hypothesis that this combination is biologically neutral in experimental models.^19^

In animal models, DRSP, unlike some androgenic progestogens, has not been associated with stimulation of breast cell proliferation, suggesting a potentially more favorable safety profile. However, the lack of robust human data, particularly regarding long-term use, underscores the need for additional clinical studies and postmarketing surveillance to confirm these findings and evaluate cancer-related safety outcomes.^19^

Only one randomized clinical trial and one cohort study evaluating BC and E4 were identified and included in this review. Both studies involved small sample sizes, precluding robust statistical analyses.

In a pilot randomized clinical trial, Singer et al.^26^ evaluated the effects of preoperative treatment with E4 20 mg vs placebo in 30 women with early-stage BC. Biopsies were obtained before treatment and during surgery at the end of treatment. To allow a comprehensive evaluation of the effects of E4 on BC cell growth and survival, the following tumor proliferation markers were assessed: Ki-67, ERα, ERβ, and apoptosis-related proteins (eg, Bax and Bcl-2). E4 demonstrated antiestrogenic effects in BC cells, including increase of apoptosis, downregulation of ERα, and decrease of systemic insulin-like growth factor 1 levels. These findings support the safety of E4 in the study setting.

The ABCE4 pilot study, published by Schmidt et al.^27^ in 2021, was a multicenter, open-label, phase IB/IIA, dose-escalation study with a 3 + 3 cohort design, in which patients with heavily pretreated advanced BC received daily oral doses of 20-, 40-, or 60-mg E4 for 12 weeks. The primary objective was to evaluate dose-limiting toxicity (DLT) and estimate the maximum recommended and optimal doses. Secondary objectives included safety, tolerability, quality of life related to estrogen-deficiency symptoms, and preliminary assessment of antitumor response. High doses of E4 were safe and well tolerated over 12 weeks, without DLT and with signs of antitumor activity in this subgroup of patients. The authors also suggested a more favorable CV risk profile than that of other estrogens, as well as the possibility of maintaining or improving quality of life.

In light of the promising results from preclinical and pilot studies, the current literature suggests that E4 may emerge as a relevant alternative in estrogen therapy (Table 2). However, this hypothesis requires confirmation in larger and more methodologically robust clinical trials.

### Venous thromboembolism

#### Hepatic and hemostatic impact and the risk of VTE

Unlike EE and E2, E4 is metabolized largely independently of cytochrome P450 enzymes and has minimal effects on the activity of these pathways. As a result, it tends to exhibit a more favorable drug-drug interaction profile than other estrogens. In the liver, E4 has a very limited impact on hepatic gene expression, which likely explains the minimal changes observed in liver function markers and coagulation parameters.^12^

In a multicenter, randomized, open-label, active-controlled study, Kobayashi et al.^28^ compared E4 15 mg/DRSP 3 mg administered in a cyclic regimen (24 days of active treatment followed by 4 hormone-free days) with EE 20 μg/DRSP 3 mg administered in an extended regimen in 86 Japanese women aged 20-50 years with endometriosis. Treatment with E4 15 mg/DRSP 3 mg produced significantly smaller increases in thrombin generation, activated protein C resistance, and D-dimer levels, as well as smaller reductions in anticoagulant proteins (free and total protein S) and free tissue factor pathway inhibitor levels. These findings suggest that the lower impact of E4/DRSP on hemostasis may represent a safer option for patients with endometriosis with regard to VTE risk.^28^

In a multicenter, randomized, double-blind, placebo-controlled phase II trial, Douxfils et al.^29^ evaluated 180 postmenopausal women aged 43-64 years who received E4 (2.5, 5, 10, or 15 mg) or placebo once daily for 12 weeks. Changes in hemostasis parameters were minimal, with a small increase only in the normalized activated protein C sensitivity ratio in the E4 15 mg group compared with placebo, suggesting a potentially favorable VTE risk profile.^29^ In another exploratory, open-label, randomized, single-center study, Douxfils et al.^30^ evaluated the effect on hemostasis of E4 15 mg/DRSP 3 mg compared with EE 30 μg/LNG 150 μg and EE 20 μg/DRSP 3 mg during six 28-day treatment cycles. Changes in hemostasis parameters after treatment with E4 15 mg/DRSP 3 mg were similar to or smaller than those observed for EE 30 μg/LNG 150 μg and EE 20 μg/DRSP 3 mg, suggesting that E4 has a lower prothrombotic effect than EE and may therefore offer greater safety with respect to VTE risk.^30^

Kluft et al.^9^ compared contraceptive combinations of E4 5 mg or 10 mg/DRSP 3 mg with EE 20 μg/DRSP 3 mg over three treatment cycles in women aged 18-35 years with a BMI between 18 and 30 kg/m^2^. Both E4/DRSP combinations had minimal effects on hemostasis parameters compared with EE 20 μg/DRSP 3 mg, suggesting a lower thrombogenic potential for E4. However, the authors emphasized that the clinical relevance of these findings should be confirmed in larger studies.^9^ It is important to note, however, that these biomarkers are not necessarily directly associated with clinical VTE outcomes, and evidence regarding a reduction in VTE risk with E4 remains limited (Table 3).^31^

## DISCUSSION

### Interpretation of current evidence

Currently available evidence suggests that E4 has a lesser impact on hepatic function and hemostatic biomarkers than other estrogens, which may translate into a lower risk of VTE without compromising contraceptive efficacy. This conclusion is based primarily on findings from phase II and III trials. However, evidence demonstrating an actual reduction in VTE risk remains insufficient.

Because VTE and BC are rare adverse events, clinical trials would require very large sample sizes and long-term follow-up to accurately estimate their true risk. Studies published to date have not shown an increased incidence of either outcome; however, they lack sufficient statistical power to establish superior or equivalent safety compared with other contraceptive options.^14^

Phase IV trials and pharmacovigilance analyses have already been published, and recent reviews (2024-2025) have discussed a lower incidence of VTE with E4. Nevertheless, the heterogeneity of the findings and the need for analyses adjusted for risk factors indicate that this issue remains unresolved. Population-based studies, national postmarketing surveillance programs with larger cohorts and longer follow-up, and clinical registries will be essential to accurately estimate comparative risk.^32,33^

The decision to use E4 should be individualized, taking into account personal and family history of BC, VTE, thrombophilia, smoking status, BMI, and age. In women at high risk of VTE or BC, other contraceptive options should be preferred, such as progestogen-only contraceptives or LNG-releasing intrauterine systems.^14,34^

## CONCLUSION

E4 stands out as a promising option among available contraceptives, exhibiting a distinct pharmacological profile characterized by limited hepatic effects and selective tissue activity. Experimental models, pharmacodynamic studies, and early clinical trials have demonstrated a lower proliferative effect on breast tissue and smaller impact on coagulation markers. However, robust long-term clinical evidence regarding the incidence of BC and VTE remains unavailable. In this context, E4 combined with DRSP emerges as a contraceptive option with a highly favorable efficacy and safety profile. Nevertheless, until more extensive follow-up data become available, it should be prescribed with the same caution applied to other oral estrogen-containing therapies in populations at high risk of VTE and BC.

## Data Availability

No research data was used.
