# Supplementary material for: Estetrol and its implications for contraception, venous thromboembolism, and breast cancer: an integrative review
Source: J Vasc Bras. 2026 Aug 28;25:e20260012. doi: 10.1590/1677-5449.20260012 (PMC13560617; doi:10.1590/1677-5449.20260012)
Supplement: Translation

# Translation: Estetrol e suas implicações na contracepção, no tromboembolismo venoso e no câncer de mama: uma revisão integrativa

**DOI:** 10.1590/1677-5449.202600121

**Authors:** Marcos Arêas Marques, Suzanna Maria Viana Sanches, Monique Magnavita Borba da Fonseca Cerqueira, Marina Araujo Zulchner, André Luiz Malavasi Longo de Oliveira

**Affiliations:** 1 Universidade Federal do Estado do Rio de Janeiro – UNIRIO, Hospital Universitário Gaffrée e Guinle, Rio de Janeiro, RJ, Brasil.; 2 Universidade do Estado do Rio de Janeiro – UERJ, Hospital Universitário Pedro Ernesto – HUPE, Rio de Janeiro, RJ, Brasil.; 3 Hospital Geral Ernesto Simões – HGES, Salvador, BA, Brasil.; 4 Universidade do Estado da Bahia – UNEB, Salvador, BA, Brasil.; 5 Universidade de São Paulo – USP, Faculdade de Medicina, São Paulo, SP, Brasil.

**Keywords:** trombose venosa profunda, embolia pulmonar, estetrol, anticoncepcionais orais combinados, câncer de mama, estrogênios

## Abstract

Os contraceptivos orais combinados (COCs) oferecem benefícios que vão além da contracepção, porém estão associados a riscos como tromboembolismo venoso (TEV) e câncer de mama. Esta revisão integrativa teve como objetivo analisar a eficácia e a segurança dos COCs contendo estetrol (E4) em relação ao risco de TEV e câncer de mama. Realizou-se uma busca na base de dados PubMed por estudos pré-clínicos e clínicos publicados até 2025. A extração dos dados seguiu roteiro estruturado, resultando na inclusão de 15 estudos nesta revisão. Evidências emergentes sugerem que o E4 exerce menor impacto hepático e sobre a cascata de coagulação, além de apresentar efeito proliferativo menos intenso no tecido mamário quando comparado a outros estrogênios. No entanto, evidências clínicas sobre desfechos duros, como incidência de TEV e câncer de mama a longo prazo, permanecem limitadas. As evidências atuais indicam um perfil promissor de eficácia e segurança, mas ainda são insuficientes para excluir os riscos de TEV e câncer de mama.

## INTRODUÇÃO

Os contraceptivos orais combinados (COCs) apresentam elevada taxa de adesão devido aos benefícios que oferecem além da contracepção, como a redução do sangramento menstrual e da dismenorreia, bem como a melhora da síndrome pré-menstrual, da acne e do hirsutismo. Adicionalmente, os COCs conferem vantagens a longo prazo, incluindo redução na incidência dos cânceres endometrial, ovariano e colorretal.

Contudo, é importante destacar que, embora raro, os COCs estão associados a efeitos adversos importantes, como o tromboembolismo venoso (TEV) e o câncer de mama^1^. O uso de COCs eleva a taxa de TEV de um valor basal de 5 casos por 10.000 mulheres/ano em não usuárias para aproximadamente 9 a 10 casos por 10.000 mulheres/ano em usuárias e varia conforme o tipo e a dose dos estrógenos e progestógenos associados na formulação. Para contextualizar esses números, vale destacar que a incidência de TEV durante a gestação é de cerca de 29 casos por 10.000 mulheres/ano, enquanto, no puerpério, esse risco aumenta para 300 a 400 casos por 10.000 mulheres/ano. Esses dados sugerem que, em determinadas situações clínicas, a contraindicação indiscriminada ao uso de COCs com base apenas na preocupação com o risco de TEV pode não ser justificada^1^.

O risco de TEV em mulheres que utilizam COCs está relacionado às alterações da hemostasia induzidas principalmente pelos estrógenos, que elevam as concentrações séricas dos fatores de coagulação (protrombina; fatores VII, VIII, X; e fibrinogênio) e reduzem os níveis dos fatores anticoagulantes (proteína S e antitrombina), além de poderem induzir resistência à proteína C ativada. As primeiras gerações de COCs, contendo doses superiores a 50 μg de etinilestradiol (EE), apresentam risco maior de TEV quando comparadas às gerações posteriores de COCs, com doses inferiores a 50 μg de EE. Entretanto, não há evidências conclusivas de redução adicional desse risco com COCs contendo 20 μg de EE quando comparados aos de 30 μg. Embora os COCs com menos de 35 μg de EE estejam associados a menor incidência de efeitos colaterais relacionados aos estrógenos, como náuseas e sensibilidade mamária nos primeiros meses de uso, essa redução não se estende ao risco de TEV^1^. Além disso, o risco também é influenciado por fatores individuais, como história pessoal ou familiar de TEV, aumento do peso corporal e da idade, trombofilia e reinício ou troca de COC após interrupção superior a 4 semanas.

Outro fator determinante para o risco de TEV é o tipo de progestógeno associado. Os progestógenos de 2^a^ geração, como levonorgestrel (LNG) e noretisterona, são considerados mais seguros em comparação aos de 3ª e 4ª gerações. Embora COCs contendo progestógenos de 3ª geração apresentem menos efeitos androgênicos e, possivelmente, menor risco cardiometabólico, podem estar associados a maior risco de TEV^1^. Por outro lado, a hemostasia não apresenta alterações significativas com contraceptivos orais exclusivos de progestógenos, implantes contendo LNG ou injeção de medroxiprogesterona em forma de depósito^1^.

O uso de COCs é um fator de risco estabelecido para câncer de mama e está associado a um discreto aumento desse risco, que retorna ao nível basal aproximadamente 10 anos após a suspensão^2^. Embora o aumento do risco individual seja modesto, o amplo uso desses métodos torna seu impacto relevante em nível populacional, correspondendo a uma estimativa de 13 casos adicionais por 100.000 mulheres/ano. No entanto, embora o aumento relativo do risco seja estatisticamente significativo, o aumento absoluto é pequeno e deve ser considerado à luz dos benefícios consolidados dos contraceptivos hormonais, como a prevenção de gestações não planejadas, associadas a maior morbimortalidade materna, e a proteção contra os cânceres de ovário e endométrio^3^.

O estetrol (E4) é um estrogênio produzido pelo fígado de feto humano durante a gestação e foi desenvolvido para uso em COCs associados à drospirenona (DRSP) e para terapia de reposição hormonal (TRH) oral isolada em mulheres com sintomas da menopausa^4^.

O objetivo desta revisão foi analisar a eficácia e a segurança do E4 na contracepção e na TRH, principalmente em relação ao seu efeito nos riscos de TEV e câncer de mama.

## MÉTODOS

Trata-se de uma revisão integrativa da literatura destinada à síntese de evidências provenientes de estudos pré-clínicos, ensaios clínicos de fases I, II e III, estudos de farmacodinâmica e análises de segurança publicados até 2025 sobre: (1) a eficácia e a segurança do E4 na contracepção; (2) os efeitos do E4 no tecido mamário e possíveis implicações para o desenvolvimento de câncer de mama; e (3) os efeitos do E4 na hemostasia e no risco de TEV.

A revisão foi conduzida em seis etapas: (1) formulação da questão de pesquisa; (2) busca e seleção dos estudos; (3) categorização; (4) avaliação metodológica; (5) interpretação dos achados; e (6) apresentação da síntese. A questão norteadora foi elaborada com base na estratégia PICO (população/problema, intervenção, comparação e desfecho [*outcome*]), resultando na pergunta: “Quais são as evidências disponíveis sobre os efeitos do E4 na contracepção, no tecido mamário e na hemostasia, incluindo suas possíveis implicações no risco de câncer de mama e de TEV?”.

A busca foi realizada na base de dados PubMed, utilizando o descritor controlado “estetrol” (MeSH e DeCS). Foram incluídos ensaios clínicos randomizados e estudos de coorte que abordavam a questão norteadora, publicados em inglês, português ou espanhol. Os resultados foram organizados em planilha Microsoft Excel^®^. A extração de dados foi feita com roteiro estruturado em quadro-síntese, contendo título, autores, população e intervenção, desenho do estudo, duração, amostra, desfechos de segurança e principais achados. Dois revisores realizaram a extração de forma independente.

## RESULTADOS

Foram identificados 282 artigos na busca bibliográfica. Após a remoção de um registro duplicado, 281 artigos foram submetidos à triagem por título e resumo. Destes, 254 foram excluídos, restando 27 estudos para avaliação do texto completo. Após a leitura integral dos artigos, 15 estudos preencheram os critérios de elegibilidade e foram incluídos na revisão (Tabelas 1, 2 e 3). O fluxograma da seleção está apresentado na Figura 1.

**Tabela 1:** Resumo dos artigos originais incluídos na revisão sobre E4 e eficácia contraceptiva.

**Autor/ano**	**Número de participantes**	**Intervenção**	**Comparador**	**Desfechos**	**Conclusão**	**Nível de evidência** ^*^
Creinin et al., 2021^4^	1.864	Multicêntrico, aberto, de fase 3: E4 15 mg + DRSP 3 mg.	—	Eficácia contraceptiva (índice de Pearl). Controle do ciclo: padrão de sangramento vaginal. Segurança: eventos adversos.	E4/DRSP foi eficaz como contraceptivo oral, com padrão de sangramento previsível para a maioria das mulheres e baixas taxas de eventos adversos.	2b
Kipling et al., 2021^5^	98	Randomizado, controlado, aberto, de três braços e paralelo (4:3:3): E4 15 mg/DRSP 3 mg *versus* EE/LNG *versus* EE/DRSP.	Comparador 1: EE 30 µg/LNG 150 µg. Comparador 2: EE 20 µg/DRSP 3 mg.	Impacto de E4 15 mg/DRSP 3 mg em parâmetros endócrinos e metabólicos após 3 e 6 meses.	E415/DRSP3 apresentou efeitos limitados em parâmetros metabólicos e endócrinos, com efeitos menos pronunciados sobre gonadotrofinas, cortisol, CBG, angiotensinogênio, SHBG e triglicerídeos quando comparado a formulações contendo EE.	1b
Gemzell‐Danielsson et al., 2021^6^	1.553	Fase 3, multicêntrico e aberto: E4/DRSP em regime 24/4 por até 13 ciclos de 28 dias.	—	1) Eficácia contraceptiva (Índice de Pearl); 2) padrões de sangramento; 3) segurança	E4/DRSP demonstrou eficácia contraceptiva, padrão de sangramento previsível e perfil de segurança favorável.	2b
Duijkers et al., 2021^7^	82	Randomizado, aberto, unicêntrico e paralelo: E4 15 mg/DRSP 3 mg (24/4) por três ciclos consecutivos.	EE 20 µg/DRSP 3 mg	Primário: supressão da função ovariana (escore de Hoogland). Secundários: função hipófise-ovariana; espessura endometrial; retorno à ovulação.	Nenhuma participante no grupo E4/DRSP ovulou e duas ovularam no grupo EE/DRSP; achados sugerem inibição adequada da ovulação e supressão da função ovariana (estudo exploratório).	1b
Apter et al., 2017^8^	396	Randomizado, aberto, multicêntrico e de definição de dose: seis ciclos (24/4) em cinco grupos: (1) E4 15 mg/DRSP 3 mg; (2) E4 15 mg/LNG 150 µg; (3) E4 20 mg/DRSP 3 mg; (4) E4 20 mg/LNG 150 µg; (5) E2V/DNG.	E2V/DNG (Qlaira^®^)	Primário: padrão de sangramento/controle de ciclo (E4+DRSP ou E4+LNG). Secundários: satisfação; bem-estar; aceitabilidade.	Neste artigo (dados reportados em publicações distintas), E4 15 mg/DRSP 3 mg associou-se a alta aceitabilidade/satisfação e perfil favorável de controle de peso corporal.	1b
Apter et al., 2016^11^	396	Mesma intervenção do estudo de definição de dose^8^; seis ciclos (24/4) em cinco grupos: (1) E4 15 mg/DRSP 3 mg; (2) E4 15 mg/LNG 150 µg; (3) E4 20 mg/DRSP 3 mg; (4) E4 20 mg/LNG 150 µg; (5) E2V/DNG.	E2V/DNG (Qlaira^®^)	Primários: padrões de sangramento vaginal e controle do ciclo.	Entre as quatro combinações com E4 avaliadas, E4 15 mg/DRSP 3 mg apresentou o perfil mais favorável de sangramento vaginal e controle do ciclo.	1b
Duijkers et al., 2015^10^	108	Piloto, de fase 2, aberto, paralelo e de avaliação de dose; três ciclos (24/4 dias) em seis grupos: (1) E4 5 mg/DRSP 3 mg; (2) E4 10 mg/DRSP 3 mg; (3) EE 20 µg/DRSP 3 mg; (4) E4 5 mg/LNG 150 µg; (5) E4 10 mg/LNG 150 µg; (6) E4 20 mg/LNG 150 µg	EE 20 µg/DRSP 3 mg	Taxas de ovulação (escore de Hoogland).	Em combinação com progestinas, E4 suprimiu adequadamente a atividade ovariana, especialmente em doses ≥ 10 mg/dia.	2b
Mawet et al., 2015^12^	109	Controlado, unicêntrico, exploratório e de avaliação de dose; três ciclos (24/4 dias) em seis grupos: (1) E4 5 mg/DRSP 3 mg; (2) E4 10 mg/DRSP 3 mg; (3) E4 5 mg/LNG 150 µg; (4) E4 10 mg/LNG 150 µg; (5) E4 20 mg/LNG 150 µg; (6) EE 20 µg/DRSP 3 mg.	EE 20 µg/DRSP 3 mg	Parâmetros hepáticos e metabólicos; marcadores ósseos; fatores de crescimento.	Combinações contendo E4 apresentaram efeitos limitados sobre função hepática, metabolismo lipídico, marcadores ósseos e parâmetros endócrinos relacionados ao crescimento em comparação a formulações com EE.	2b
Kluft et al., 2016^9^	48	Aberto, unicêntrico, paralelo e de definição de dose; regime 24/4 em três grupos: (1) EE 20 µg/DRSP 3 mg (Yaz®); (2) E4 5 mg/DRSP 3 mg; (3) E4 10 mg/DRSP 3 mg.	EE 20 µg/DRSP 3 mg (Yaz^®^)	Marcadores de estrogenicidade hepática e hemostasia.	Achados sugerem menor estrogenicidade hepática de E4/DRSP *versus* EE/DRSP, gerando a hipótese de menor risco potencial de TEV.	2b

E4 = estetrol; DRSP = drospirenona; EE = etinilestradiol; LNG = levonorgestrel; E2V = valerato de estradiol; DNG = dienogeste; CBG = globulina ligadora de corticosteroides; SHBG = globulina ligadora de hormônios sexuais; TEV = tromboembolismo venoso. Regime 24/4 = 24 dias de comprimidos ativos e 4 dias de placebo/intervalo sem hormônio.

* Níveis de Evidência do Oxford Centre for Evidence-Based Medicine (OCEBM), versão de março de 2009.

**Tabela 2:** Resumo dos artigos originais incluídos na revisão sobre E4 e segurança relacionada ao câncer de mama.

**Autor/ ano**	**N**	**Intervenção**	**Comparador**	**Desfechos**	**Conclusão**	**Nível de evidência** ^*^
Singer et al., 2014^26^	30	RCT piloto: E4 20 mg *versus* placebo no perioperatório de câncer de mama em estágio inicial; biópsias pré- e pós-operatórias.	Placebo	Marcadores de proliferação tumoral e apoptose: Ki-67, REα, REβ, Bax, Bcl-2; níveis sistêmicos de IGF-1.	E4 apresentou efeitos antiestrogênicos em células de câncer de mama, com aumento de apoptose, redução da expressão de REα e diminuição dos níveis sistêmicos de IGF-1, sugerindo segurança do E4 no contexto avaliado.	1b
Schmidt et al., 2021^27^	12	Estudo piloto multicêntrico, aberto, fase IB/IIA, escalonamento de dose (coorte 3+3): E4 20/40/60 mg VO diária por 12 semanas em câncer de mama avançado (*heavily pretreated*).	—	Primário: TLD e estimativa de dose máxima recomendada/ideal. Secundários: segurança, tolerabilidade, qualidade de vida relacionada a sintomas de deficiência estrogênica, resposta antitumoral preliminar.	Doses elevadas de E4 foram descritas como seguras e bem toleradas por 12 semanas, com sinais de efeito antitumoral e ausência de TLD, sugerindo perfil de risco cardiovascular potencialmente favorável e possibilidade de manutenção/melhora da qualidade de vida.	4

E4 = estetrol; TLD = toxicidade limitante da dose; REα = receptor de estrogênio α; REβ = receptor de estrogênio β; VO = via oral; IGF-1 = fator de crescimento semelhante à insulina-1; RCT = ensaio clínico randomizado e controlado.

* Níveis de Evidência do Oxford Centre for Evidence-Based Medicine (OCEBM), versão de março de 2009.

**Tabela 3:** Resumo dos artigos originais incluídos na revisão sobre E4 e segurança relacionada ao TEV.

**Autor/ano**	**Número de participantes**	**Intervenção**	**Comparador**	**Desfechos**	**Conclusão**	**Nível de evidência** ^*^
Douxfils et al., 2020^30^	101	Estudo clínico randomizado: uso de contraceptivo oral combinado contendo E4 15 mg + DRSP 3 mg por seis ciclos de 28 dias em mulheres saudáveis.	EE 20 μg + DRSP 3 mg	Parâmetros de hemostasia, incluindo resistência à proteína C ativada, fragmento 1+2 da protrombina, SHBG e outros marcadores associados ao risco tromboembólico, avaliados após 3 e 6 ciclos.	O E4/DRSP apresentou impacto menor ou semelhante nos marcadores de hemostasia quando comparado ao EE/LNG e menor impacto em relação ao EE/DRSP, sugerindo um perfil hemostático mais favorável. Os achados indicam possível redução do risco tromboembólico associado ao componente estrogênico.	1b
Kluft et al., 2017^9^	48	Estudo clínico aberto: E4 (5 ou 10 mg) + DRSP 3 mg por três ciclos em mulheres saudáveis.	EE 20 μg + DRSP 3 mg	Marcadores de hemostasia e estrogenicidade hepática (SHBG, angiotensinogênio, APCr, D-dímero, F1+2).	E4/DRSP apresentou menor impacto hemostático e ausência de efeito pró-coagulante em comparação ao EE/DRSP, sugerindo perfil trombótico potencialmente mais favorável.	2b
Kobayashi et al., 2024^28^	86	Estudo randomizado multicêntrico: E4 15 mg + drospirenona 3 mg por 12 semanas em pacientes com endometriose.	EE 20 μg + DRSP 3 mg	Marcadores de coagulação e fibrinólise (APCsr/nAPCsr, D-dímero, F1+2, proteína S, TFPI, SHBG).	E4/DRSP apresentou menor impacto nos sistemas de coagulação e fibrinólise em comparação ao EE/DRSP, sugerindo perfil trombótico mais favorável, inclusive em população com maior risco basal de TEV.	1b
Douxfils et al., 2023^29^	180	Estudo randomizado duplo-cego: E4 2,5-15 mg/dia por 12 semanas.	Placebo	Marcadores de hemostasia (nAPCsr, D-dímero, F1+2), SHBG, perfil lipídico, metabolismo glicídico e marcadores de remodelação óssea.	O E4 apresentou impacto mínimo na hemostasia, com alterações discretas e clinicamente irrelevantes, além de efeitos metabólicos e ósseos potencialmente benéficos, sugerindo perfil trombótico favorável em mulheres pós-menopáusicas.	1b

APCsr = razão de sensibilidade à proteína C ativada; DRSP = drospirenona; E4 = estetrol; EE = etinilestradiol; LNG = levonorgestrel; nAPCsr = razão de sensibilidade à proteína C ativada normalizada; SHBG = globulina ligadora de hormônios sexuais; TEV = tromboembolismo venoso.

* Níveis de Evidência do Oxford Centre for Evidence-Based Medicine (OCEBM), versão de março de 2009.

**Figura 1:** Fluxograma de seleção de estudos incluídos. TEV = tromboembolismo venoso.

### Eficácia e segurança na contracepção

Foram identificados nove estudos sobre o uso do E4 na contracepção, incluindo estudos de coorte e ensaios clínicos randomizados^4-12^.

Em estudos iniciais de fase II^8-12^, foram avaliadas diferentes doses de E4 (5, 10, 15 e 20 mg) em associação com DRSP 3 mg e LNG 150 µg, com foco em supressão ovariana, padrões de sangramento, aceitabilidade e parâmetros metabólicos/hepáticos. Para fins comparativos, algumas investigações incluíram formulações contendo valerato de estradiol (E2V) associado a dienogeste (DNG) e EE 20 µg associado a DRSP 3 mg. Em um estudo piloto, doses de E4 acima de 10 mg, quando combinadas com progestina, demonstraram supressão ovariana adequada^10^.

A combinação E4 15 mg/DRSP 3 mg apresentou parâmetros positivos de aceitabilidade e satisfação em usuárias, associando-se também a um perfil favorável de controle ponderal. Em análise comparativa entre diferentes combinações contendo E4, a combinação E4 15 mg/DRSP 3 mg foi descrita como a formulação com o perfil de sangramento vaginal e controle de ciclo mais favorável^8,11^.

No estudo multicêntrico, exploratório e aberto FIESTA, 396 mulheres saudáveis com idades entre 18 e 35 anos foram alocadas em cinco grupos de tratamento e acompanhadas por seis ciclos: (1) E4 15 mg/DRSP 3 mg; (2) E4 20 mg/DRSP 3 mg; (3) E4 15 mg/LNG 150 µg; (4) E4 20 mg/LNG 150 µg; e (5) E2V/DNG (Qlaira**^®^**; comparador). Os resultados do ensaio foram relatados em duas publicações^8-11^, as quais consistentemente demonstraram que a combinação E4 15 mg/DRSP 3 mg apresentou alta aceitabilidade e satisfação, além de um perfil favorável para controle ponderal e um padrão de sangramento mais vantajoso em comparação às demais combinações avaliadas.

Em um estudo randomizado, aberto e de centro único, 82 participantes foram alocadas na razão 1:1 para receber E4 15 mg/DRSP 3 mg ou EE 20 µg/DRSP 3 mg por três ciclos consecutivos. Foram avaliadas a supressão da função ovariana (expressa pelo escore de Hoogland), a função hipófise-ovariana, a espessura endometrial e o retorno da ovulação. Na análise exploratória, não houve ovulação no braço E4 15 mg/DRSP 3 mg, enquanto duas participantes ovularam no braço EE 20 µg/DRSP 3 mg (uma delas em duas ocasiões no mesmo ciclo), sugerindo supressão ovulatória adequada com E4 15 mg/DRSP 3 mg^7^.

Outro estudo aberto, controlado e de desenho paralelo randomizou 98 participantes na razão 4:3:3 para três braços de tratamento: (1) E4 15 mg/DRSP 3 mg; (2) EE 30 µg/LNG 150 µg; e (3) EE 20 µg/DRSP 3 mg. Foram avaliados parâmetros endócrinos e metabólicos após três e seis ciclos, incluindo gonadotrofinas, globulina ligadora de hormônios sexuais, cortisol, globulina ligadora de cortisol, angiotensinogênio e triglicerídeos, entre outros. Em comparação com as formulações contendo EE, a formulação E4 15 mg/DRSP 3 apresentou apenas efeitos limitados sobre esses parâmetros^5^.

Com base nesses dados, a dose de E4 15 mg/DRSP 3 mg foi escolhida para investigação em dois ensaios clínicos pivotais de fase III (E4 FREEDOM), conduzidos na Europa/Rússia e nos Estados Unidos/Canadá. Os estudos avaliaram a eficácia contraceptiva (índice de Pearl), o controle de ciclo (padrão de sangramento de acordo com os critérios de Mishell) e a segurança^4,6^.

O estudo conduzido nos Estados Unidos/Canadá foi realizado em 77 centros e incluiu 1.864 participantes com idades entre 16 e 50 anos e índice de massa corporal (IMC) ≤ 30 kg/m^2^, que utilizaram E4 15 mg/DRSP 3 mg durante 13 ciclos (regime 24/4). A eficácia contraceptiva foi avaliada pelo índice de Pearl e pela falha do método; o controle do ciclo foi avaliado pelo padrão de sangramento; e a segurança foi descrita por eventos adversos. Foram reportados índice de Pearl de 2,65 (IC95%, 1,73-3,88) e índice de falha do método de 1,43 (IC95%, 0,70-2,39). Sangramento programado foi observado em 82,9 a 87,0% das participantes por ciclo, com duração média de 4,5 dias, e houve redução de sangramentos não programados após os ciclos iniciais. Os eventos adversos mais frequentes foram cefaleia (5,0%) e metrorragia (4,6%), sem TEV relatado na população estudada^4^.

O estudo conduzido na Europa/Rússia foi realizado em 69 centros e incluiu 1.553 participantes com idades entre 18 e 50 anos e IMC ≤ 35 kg/m^2^, que utilizaram E4/DRSP durante 13 ciclos (regime 24/4). O desfecho primário foi a eficácia contraceptiva (índice de Pearl) na subpopulação de 18 a 35 anos; desfechos secundários incluíram falha do método, taxas cumulativas de gravidez, padrões de sangramento e segurança. Foi reportado um índice de Pearl de 0,47 gestações por 100 mulheres/ano (IC95% 0,15-1,11) e índice de falha do método de 0,29 gestações por 100 mulheres/ano (IC95% 0,06-0,83) na subpopulação de 18 a 35 anos. Sangramento programado/*spotting* ocorreu em 91,9 a 94,4% das participantes por ciclo, com duração média de 4 a 5 dias, e sangramentos não programados reduziram ao longo do tratamento (de 23,5% para < 16% em 6 meses). Os eventos adversos mais frequentes foram cefaleia (7,7%), metrorragia (5,5%), hemorragia vaginal (4,8%) e acne (4,2%) (Tabela 1)^6^.

Análises agrupadas derivadas dos ensaios clínicos de fase III^13-16^ não foram incluídas no corpo de evidências considerado nesta revisão integrativa, por representarem análises secundárias de populações já descritas em estudos previamente publicados. No entanto, essas análises fornecem informações complementares relevantes sobre subgrupos e desfechos clinicamente importantes, incluindo tolerabilidade, padrões de sangramento, segurança e eficácia em populações específicas, razão pela qual seus principais achados são brevemente apresentados a seguir.

Em uma avaliação de participantes com fatores de risco cardiovasculares, não foram observadas diferenças significativas nas taxas de descontinuação entre aquelas com e sem fatores de risco. Entre mais de 1.400 usuárias com fatores de risco, apenas 0,2% descontinuaram por hipertensão arterial sistêmica, sendo que todas apresentavam níveis tensionais limítrofes e pelo menos um outro fator de risco cardiovascular^13^.

Uma análise agrupada de 3.417 participantes dos ensaios clínicos de fase III revelou tolerabilidade favorável e ausência de diferenças clinicamente relevantes em peso, pressão arterial, frequência cardíaca e parâmetros laboratoriais durante o tratamento^14^. Uma análise de eficácia em subgrupos confirmou a alta efetividade global, associando falhas contraceptivas a fatores predominantemente não modificáveis (exceto aderência), como raça negra, menor idade e gestação prévia^15^. Por fim, em uma avaliação do padrão de sangramento em 3.409 participantes, 87,2 a 90,4% apresentaram sangramento programado/*spotting* por ciclo, caracterizando padrão previsível. A aderência e o IMC influenciaram esses desfechos^16^ (Tabela 1).

### E4 e câncer de mama

Os efeitos dos estrogênios são mediados principalmente pelos receptores de estrogênio α (REα) e β (REβ), que atuam como fatores de transcrição na regulação de genes-alvo por meio da via genômica. Além disso, os estrogênios podem desencadear respostas rápidas ao ativar a sinalização iniciada pela membrana, via receptor de estrogênio acoplado à proteína G (GPER, do inglês *G protein-coupled estrogen receptor*), presente em células normais e neoplásicas, incluindo as do câncer de mama^17^. A sinalização iniciada pelo GPER pode induzir a transcrição de genes envolvidos em processos como proliferação celular, invasão, metástase, angiogênese e inflamação promotora de neoplasias^17^.

Os possíveis benefícios do uso do E4 no desfecho “câncer de mama” podem ser considerados em dois contextos: (1) como componente da TRH na menopausa, com potencial perfil de risco neoplásico mais favorável; e (2) como possível estratégia terapêutica em câncer de mama já estabelecido, com base em efeitos antiestrogênicos observados em determinadas condições experimentais.

A literatura atual indica aumento do risco de câncer de mama associado à TRH, sobretudo quando há combinação de estrógeno com progestógeno, sugerindo maior impacto sobre células neoplásicas preexistentes do que sobre a gênese tumoral propriamente dita^18-19^. De forma semelhante, COCs estão associados a um discreto aumento desse risco, com tendência de retorno ao nível basal aproximadamente 10 anos após a sua descontinuação, o que também é compatível com a hipótese de efeito predominante sobre lesões preexistentes^19^.

O E4 apresenta afinidade pelo REα e pelo REβ, mas com comportamento peculiar quando comparado a outros estrógenos, sendo sua afinidade pelo REα quatro a cinco vezes maior. Por essa característica, é classificado como estrogênio nativo com atividade tecidual seletiva (*native estrogen with selective tissue activity*, em inglês)^20^, atuando como antagonista do REα na membrana celular e como agonista nos receptores nucleares, resultando em efeitos predominantemente pró-estrogênicos nos ossos, no endométrio, na vagina e no sistema cardiovascular, além de efeitos antiestrogênicos no tecido mamário. Esse conjunto de características sustenta a hipótese de que o E4 possa atuar como modulador seletivo do receptor de estrogênio (*selective estrogen receptor modulator*, em inglês), apoiada por evidências *in vitro* e *in vivo*, além de apresentar afinidade de ligação e potência estrogênica inferiores às do 17β-estradiol (E2)^21^.

Em estudos experimentais, o E4 associou-se a uma menor proliferação de células mamárias em comparação ao E2, principal estrogênio biologicamente ativo em mulheres na fase reprodutiva. Em modelos de câncer de mama RE-positivo, o E4 apresentou ações mistas (agonistas e antagonistas), com capacidade de antagonizar a proliferação e a migração celular induzidas pelo E2. O E4 também demonstrou propriedades pró-apoptóticas em células com resistência endócrina, ativação diferencial de vias de sinalização, menor indução de genes proliferativos e efeitos anti-invasivos mediados por GPER e pelo inibidor do ativador do plasminogênio-2 (*plasminogen activator inhibitor type 2*, em inglês)^18,22^. Esses achados sustentam a relevância do E4 nas discussões sobre terapia estrogênica em populações de risco e em pacientes com câncer de mama.

Os efeitos do E4, entretanto, parecem ser dependentes da dose. Em concentrações elevadas, foram descritos efeitos pró-tumorais e antiapoptóticos em células estrogênio-sensíveis, ainda que com potência inferior à do E2^23^. Doses supraterapêuticas, superiores a 10 vezes as utilizadas em estudos de contracepção e menopausa, produziram efeitos semelhantes aos do E2 terapêutico em células mamárias, o que, ainda assim, tem sido interpretado como compatível com um perfil de segurança potencialmente mais favorável do E4 em condições de uso clínico habitual^18,21^.

Estudos experimentais realizados desde a década de 1980 sugerem que o E4 é 50 a 100 vezes menos potente que o E2 na indução de receptores de progesterona, exibindo propriedades agonistas e antagonistas em condições distintas. Trabalhos posteriores indicam que E1, E2, E3 e E4 podem estimular proliferação tumoral em altas concentrações. Contudo, em doses baixas, o E4 apresenta efeito significativamente menor que o E2. A associação de E2 com E4 foi avaliada em modelos experimentais, sugerindo neutralização parcial dos efeitos proliferativos do E2 quando combinado ao E4 em determinadas condições, com interpretação de ação antiestrogênica, embora com mecanismos ainda não completamente elucidados. Adicionalmente, estudos *in vitro* e *in vivo* descrevem menor impacto do E4 sobre a motilidade e invasão celular^18,23-25^.

A combinação de E4 com progestógeno, proposta como estratégia de proteção endometrial, foi avaliada em estudos pré-clínicos, com resultados sugerindo efeito globalmente neutro sobre o crescimento e a disseminação de câncer de mama já instalado. Nesse conjunto de dados, o E4 apresenta perfil mais favorável do que o E2, e as combinações com progestógenos não demonstraram aumento evidente de atividade pró-tumoral. Análises transcriptômicas também sugerem impacto transcricional limitado da associação de E4 com progestógeno sobre processos relacionados ao crescimento mamário, reforçando a hipótese de neutralidade biológica dessa combinação em modelos experimentais^19^.

Em modelos animais, a DRSP, diferentemente de progestógenos com atividade androgênica, não foi associada a estímulo de proliferação mamária, sugerindo um potencial perfil de segurança mais favorável. Entretanto, a ausência de dados robustos em humanos, particularmente quanto ao uso prolongado, sustenta a necessidade de estudos clínicos adicionais e de seguimento pós-comercialização para a confirmação desses achados e a avaliação de desfechos de segurança oncológica^19^.

Apenas um ensaio clínico randomizado e um estudo de coorte avaliando E4 em relação ao câncer de mama foram identificados e incluídos nesta revisão. Ambos apresentaram tamanho amostral reduzido, o que limitou a realização de análises estatísticas robustas.

Em um ensaio clínico randomizado piloto, Singer et al.^26^ avaliaram os efeitos do tratamento pré-operatório com E4 20 mg *versus* placebo em 30 mulheres com câncer de mama em estágio inicial. Foram realizadas biópsias antes do início do tratamento e durante o procedimento cirúrgico, ao final da intervenção. Para permitir uma avaliação abrangente do impacto do E4 sobre o crescimento e a sobrevivência celulares, foram analisados os seguintes marcadores de proliferação tumoral: Ki-67, REα, REβ e proteínas relacionadas à apoptose (por exemplo, Bax e Bcl-2). O E4 demonstrou efeitos antiestrogênicos em células de câncer de mama, com aumento de apoptose, redução da expressão de REα e diminuição dos níveis sistêmicos de fator de crescimento semelhante à insulina-1 (*insulin-like growth factor 1*, em inglês), achados que sugerem segurança do E4 no contexto avaliado.

Adicionalmente, o estudo piloto ABCE4, publicado por Schmidt et al.^27^ em 2021, foi um estudo multicêntrico, aberto, fase IB/IIA, de escalonamento de dose, com desenho de coorte 3+3, no qual pacientes com câncer de mama avançado, previamente submetidas a múltiplas linhas terapêuticas (*heavily pretreated*), receberam doses orais diárias de E4 de 20, 40 ou 60 mg por 12 semanas. O objetivo primário foi avaliar toxicidade limitante de dose (TLD) e estimar a dose máxima recomendada e a dose ideal. Entre os objetivos secundários, incluíram-se segurança, tolerabilidade, qualidade de vida relacionada a sintomas de deficiência estrogênica e resposta antitumoral preliminar. Os autores concluíram que doses elevadas de E4 foram seguras e bem toleradas ao longo de 12 semanas, com sinais de atividade antitumoral nesse subgrupo, sem ocorrência de TLD, sugerindo ainda um perfil cardiovascular potencialmente mais favorável em comparação a outros estrogênios e a possibilidade de manutenção ou melhora da qualidade de vida.

Diante de resultados promissores em estudos pré-clínicos e pilotos, a literatura sugere que o E4 pode emergir como uma alternativa relevante na terapia estrogênica (Tabela 2). Entretanto, essa hipótese requer confirmação em estudos clínicos maiores e metodologicamente mais robustos.

### Tromboembolismo venoso

#### Impacto hepático, hemostático e o risco de TEV

Diferentemente do EE e do E2, o E4 praticamente não depende das enzimas do citocromo P450 para sua metabolização, interferindo minimamente na atividade dessas vias. Como resultado, tende a apresentar um perfil de interações medicamentosas mais favorável em comparação a outros estrogênios. No fígado, o E4 exerce impacto muito discreto sobre a expressão gênica hepática, o que provavelmente explica as alterações mínimas em marcadores de função hepática e nos parâmetros de coagulação^12^.

Em um estudo multicêntrico, randomizado, aberto e com controle ativo, Kobayashi et al.^28^ avaliaram 86 mulheres japonesas com endometriose, com idades entre 20 e 50 anos, comparando E4 15 mg/DRSP 3 mg em regime cíclico (24 dias com hormônio e 4 dias sem) com EE 20 µg/DRSP 3 mg em regime contínuo estendido. Em comparação com EE 20 µg/DRSP 3 mg, a combinação E4 15 mg/DRSP 3 mg esteve associada a aumentos significativamente menores na geração de trombina, na resistência à proteína C ativada e nos níveis de D-dímero, bem como a reduções menos acentuadas dos níveis de proteínas anticoagulantes (proteína S livre e total) e do inibidor da via do fator tecidual (livre). Esses achados sugerem que o menor impacto dessa combinação sobre a hemostasia pode representar uma opção mais segura em pacientes portadoras de endometriose em relação ao risco de TEV^28^.

Em um estudo de fase II multicêntrico, randomizado, duplo-cego e controlado por placebo, Douxfils et al.^29^ avaliaram 180 mulheres na pós-menopausa, com 43-64 anos de idade, que receberam tratamento com E4 (2,5, 5, 10 e 15 mg) ou placebo uma vez ao dia por 12 semanas. Foram observadas alterações mínimas nos parâmetros de hemostasia, com um pequeno aumento apenas na razão de sensibilidade à proteína C ativada normalizada no grupo E4 15 mg em comparação ao placebo, o que pode representar um perfil de segurança favorável em relação ao risco de TEV^29^. Em outro estudo exploratório, aberto, randomizado e unicêntrico, Douxfils et al.^30^ compararam os efeitos de E4 15 mg/DRSP 3 mg, EE 30 µg/LNG 150 µg e EE 20 µg/DRSP 3 mg sobre parâmetros hemostáticos durante seis ciclos de 28 dias. A combinação E4 15 mg/DRSP 3 mg apresentou impacto hemostático semelhante ou menor que o observado com EE 30 µg/LNG 150 µg e EE 20 µg/DRSP 3 mg, corroborando a hipótese de um menor efeito pró-trombótico do E4 em relação ao EE e, consequentemente, de maior segurança em relação ao risco de TEV^30^.

Kluft et al.^9^ compararam as combinações contraceptivas E4 5 mg/DRSP 3 mg e E4 10 mg/DRSP 3 mg com EE 20 µg/DRSP 3 mg durante três ciclos de tratamento em mulheres com idades entre 18 e 35 anos e IMC entre 18 e 30 kg/m^2^. As duas combinações de E4/DRSP tiveram impacto mínimo na hemostasia quando comparadas à combinação EE 20 µg/DRSP 3 mg, o que conferiria um menor potencial trombogênico ao E4, com a ressalva de que o impacto clínico desse achado deveria ser validado por estudos maiores^9^. No entanto, é importante ressaltar que esses biomarcadores não estão necessariamente diretamente associados aos desfechos clínicos de TEV, e as evidências sobre sua redução com o uso do E4 permanecem limitadas (Tabela 3)^31^.

## DISCUSSÃO

### Interpretação dos dados atuais

Os dados publicados até o momento sugerem, de fato, uma redução de efeitos hepáticos e um impacto mais brando sobre biomarcadores da hemostasia com o uso do E4 em comparação a outros estrogênios, o que potencialmente reduziria o risco de TEV sem comprometer a eficácia, com base principalmente em estudos de fase II e III. No entanto, as evidências de redução real do risco de TEV ainda são insuficientes.

Pelo fato de o TEV e o câncer de mama serem eventos adversos raros, os ensaios clínicos exigiriam amostras muito amplas e seguimento prolongado para estimar o risco real. As séries publicadas até agora não evidenciaram aumento da incidência desses eventos; no entanto, não possuem poder estatístico suficiente para confirmar segurança superior ou equivalente às demais alternativas de contracepção^14^.

Há estudos de fase IV e análises de farmacovigilância disponíveis, bem como revisões recentes (2024-2025) que discutem a menor incidência de TEV com o E4. Contudo, a heterogeneidade dos resultados e a necessidade de análises ajustadas por fatores de risco tornam essa questão ainda indefinida. Estudos populacionais e nacionais de vigilância pós-comercialização, com coortes maiores e seguimento mais longos, e registros clínicos serão imprescindíveis para uma estimativa precisa do risco comparativo^32,33^.

A decisão sobre o uso de E4 deve ser individualizada, considerando o histórico pessoal e familiar de câncer de mama, TEV, trombofilia, tabagismo, IMC e idade. Em mulheres com alto risco de TEV ou de câncer de mama, outras opções contraceptivas ainda devem ser consideradas prioritárias, como progestógenos isolados ou sistemas intrauterinos liberadores de LNG^14,34^.

## CONCLUSÃO

O E4 destaca-se como opção promissora no arsenal de contraceptivos, apresentando um perfil farmacológico diferenciado, com menor impacto hepático e efeitos tissulares seletivos. Em modelos experimentais, estudos farmacodinâmicos e ensaios clínicos iniciais, observaram-se menor risco proliferativo mamário e menor alteração de marcadores de coagulação. Entretanto, evidências clínicas robustas a longo prazo sobre a incidência de câncer de mama e TEV ainda não estão disponíveis. Nesse contexto, a combinação E4/DRSP emerge como uma opção com perfil de eficácia e segurança muito interessante, mas que ainda deve ser avaliada com as mesmas precauções aplicadas às demais terapias estrogênicas orais em populações de alto risco de TEV e câncer de mama, até que dados de seguimento mais extensos estejam consolidados.

## Data Availability

Nenhum dado de pesquisa foi utilizado.
